# Using Acoustic Vibrations as a Method for Implant Insertion Assessment in Total Hip Arthroplasty

**DOI:** 10.3390/s22041609

**Published:** 2022-02-18

**Authors:** Jonathan C. J. Wei, Willem H. A. Crezee, Hilda Jongeneel, Tobias S. A. De Haas, Wesley L. A. Kool, Bryan J. Blaauw, Jenny Dankelman, Tim Horeman

**Affiliations:** 1 Department of BioMechanical Engineering, Faculty of Mechanical, Maritime and Materials Engineering, Delft University of Technology, 2628 CD Delft, The Netherlands; w.h.a.crezee@student.tudelft.nl (W.H.A.C.); h.jongeneel@student.tudelft.nl (H.J.); t.s.a.dehaas@student.tudelft.nl (T.S.A.D.H.); w.l.a.kool@student.tudelft.nl (W.L.A.K.); j.dankelman@tudelft.nl (J.D.); t.horeman@tudelft.nl (T.H.); 2 Department of Orthopaedics, Elkerliek Hospital, 5707 HA Helmond, The Netherlands; bjblaauw@me.com

**Keywords:** medical device, surgery, hip arthroplasty, acoustics, vibration emissions

## Abstract

The success of total hip arthroplasty depends on the experience of the surgeon, and one of the ways the surgeon currently determines the final implant insertion depth is to listen to the change in audible pitch of the hammering sound. We investigated the use of vibration emissions as a novel method for insertion quality assessment. A non-invasive contact microphone-based measurement system for insertion depth estimation, fixation and fracture detection was developed using a simplified in vitro bone/implant (n = 5). A total of 2583 audio recordings were analyzed in vitro to obtain energy spectral density functions. Out of the four main resonant peaks under in vitro conditions, broach insertion depth statistically correlates to increasing 3rd and 4th peak frequencies. Degree of fixation was also observed as higher goodness of fit (0.26–0.78 vs. 0.12–0.51 between two broach sizes, the latter undersized). Finally, however, the moment of fracture could not be predicted. A cadaveric in situ pilot study suggests comparable resonant frequencies in the same order of magnitudes with the bone model. Further understanding of the signal patterns are needed for an early warning system diagnostic system for imminent fractures, bone damage, improving accuracy and quality of future procedures.

## 1. Introduction

An ever-increasing number of people are receiving total hip arthroplasties (THAs) in recent years. In the Netherlands, this number grew from 23,000 to 41,000 between 2010 and 2018 [1] and is predicted to increase even further due to population growth and increase in life expectancy. When a total hip arthroplasty (THA) is performed, the head of the femur is removed and a broach is inserted into the femur by hammering, to provide a canal, in which the stem can be press-fitted. Over-insertion of the broach or stem may cause fractures [2] and under-insertion does not provide sufficient stability to the implant. To prevent this, it is crucial to detect the insertion depth “sweet spot” where the fixation is sufficient and the risk of fracture is minimal, defined herein as the ‘end point’ of insertion. In addition to the stability of the implant, surgeons also fine-tune this end point to achieve equal limb length using audible pitch changes of the hammering sound. Thus, the ability to control the insertion distance would yield higher accuracy and stability [3]. Therefore, the experience of the surgeon is critical for the sufficient implant fixation and the localization of this end point [4], and the success of the surgery.

At present, the fixation end point is determined by the surgeon with leg length equality in mind and is adjusted several times during surgery, often by eye, by comparing the operated side with the non-operated side. This adjustment is performed by hammering the broach to the desired depth, or a broach of the next size is used if stability is not deemed sufficient. Extra attention should be given to a potentially increased risk of fracture. If the press-fit is overloaded, then it can risk bone fracture. This relationship is difficult to determine, so we propose herein a method and a device that could help make that determination.

In recent years, several studies have examined what can be achieved with Acoustic Emission (AE) and vibration analysis in orthopedics [5] for wear [6], aseptic loosening [7], microdamage to bone structures, healing, and biomechanical environment assessment with free or forced vibrations [4,8]. Some of these studies, however, focused on using acoustic emissions as a diagnostic tool over long periods of time to assess fixation quality and wear (sometimes through the skin of patients with THA [6,9]), rather than real-time analysis of implant insertion quality intraoperatively. There are, however, a few studies that explored the effects of acoustic emissions as a tool for fixation, microcracking or fracture prediction during joint replacements in various models such as animal [10] and human cadaver [8]; in vitro [11,12] and in vivo setups [3,4] with various degrees of success.

Several other techniques of measuring AEs were also trialed. For example, Pastrav et al. [4] studied the frequency response function (FRF) change of the stem–femur system with an electrodynamic exciter (shaker) to excite low amplitude vibrations in the stem over hammer blows to detect the insertion end point. They also suggested [13] that intraoperative vibration analysis requires a more user-friendly device. Oberst et al. [8] used a microphone aimed at the hammer impaction point. Pechon et al. [10] used an acoustic emission sensor placed on the stem inserted into deer femora and placed in a loading machine (quasistatically-loaded instead of striking with a hammer). These studies suggested a correlation between acoustic emission signals and occurring damage processes. Shibunama et al. [14] used a contact microphone on the bone itself (n = 1) to pick up less ambient noise and suggested that their system can help determine the right stem size. Other similar arthroplasties involving acoustic analysis have been carried out recently in an in vitro femur setup [15,16], acetabulum setup (with force sensors) [17], (with non-contact audio microphone) [18], in silico femur setup [16] and in vivo [19].

The types of bone setups for acoustic emission investigations carried out for THAs in the present literature, include sounds directly obtained from the OR [20], cadaver bone [8], animal bone [10], artificial bone [11] or a simplified bone model [12]. To design an appropriate bone model, some bone characteristics are required. Cortical and trabecular bone differ in elastic modulus (11,000–20,000 to 5–150 MPa, respectively) as reported by Kutz [21], and furthermore, the cortical bone is an anisotropic material (about twice the longitudinal strength to transversal strength). In THA fracture experiments, the ultimate tensile strength of the bone model in the transverse direction plays a considerable part in determining the fracture resistance of the material.

While the above investigations appear to be promising, many have been tested in controlled settings not directly applicable to surgical settings, furthermore, it is often obstructive, with complex audio recording and processing hardware such as microphones, wiring, as well as interference from ambient sounds etc. Mulier et al. [13] emphasized that while “in vivo use of vibration analysis is possible, there is an urgent need for the development of a more user-friendly, wireless device”.

Another aspect of measuring AEs to consider is that impact force while broaching is not constant since it depends on both the surgeon and the impact duration. According to Oberst et al. [8], it can vary between 1 and 19 kN, with a mode of approximately 3.5 kN. Crisman et al. [22] showed a typical impact force plot with a maximum force of 7.6 kN, decreasing to zero in approximately 1 ms. Combing these results, by integrating over time, the impact energy, which is probably more consistent than the impact force, is between 0.5–9.5 J per hammer blow.

The aim of the study is to develop and validate a simple, non-invasive vibration measurement system as a potential tool for estimating insertion depth, fixation, and fracture development, that does not jeopardize the current workflow. This novel, non-invasive Broach Insertion Measurement System (BIMS) is designed and tested in vitro by analyzing the frequency spectrum of the hammering impact sounds in a systematic way.

With BIMS, we test the following hypotheses:

1. The change in measured vibration peak frequencies is linked to the insertion depth in the bone model;
2. The degree of fixation of the broach in the bone affects the convergence of peak energies at peak frequencies;
3. The peak frequencies of the hammer impact sound can be used to predict imminent fracture.

## 2. Materials and Methods

### 2.1. Broach Insertion Measurement System (BIMS)

#### 2.1.1. Microphone Selection

**Figure 1:**
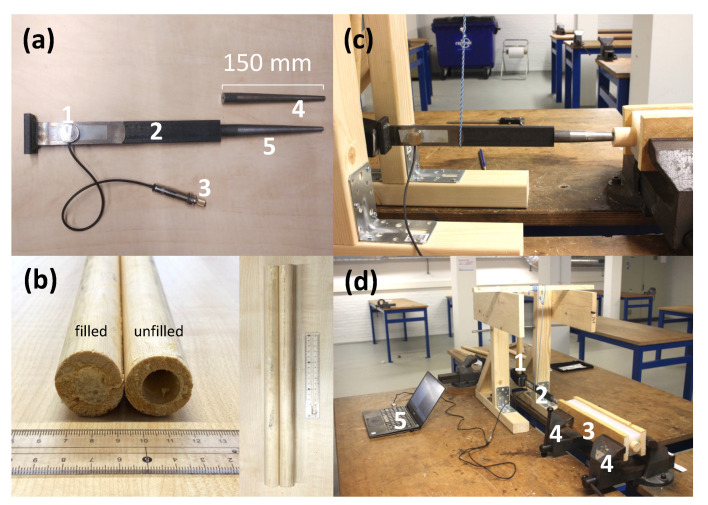
(**a**) The simplified implant model consisting of a contact microphone **1**, broach handle **2**, a 6.3 mm jack to the computer **3**, smaller broach **4** and a larger broach **5**. (**b**) Example of filled and unfilled bone model. Experiment setup (**c**) side view and overview (**d**) showing the swing hammer **1**, hammer and broach model with the attached microphone **2**, bone in silicone frame **3** clamped between two vices **4** and the computer to record and process the data **5**.

In order to analyze the vibration produced by hammering, the sound was recorded with a piezo contact microphone (Oyster S/S, Schaller, Postbauwer-Heng, Germany) with a pickup capacity of 74 pF placed directly on the broach handle. The piezo element is capable of picking up frequencies in the audible spectrum. The microphone was attached to the handle with double-sided tape (double-sided filmic tape, Tesa, Norderstedt, Germany), just above the place where the surgeon holds the handle, shown in Figure 1a. This enables the signal to be directly transmitted without having to travel through air.

#### 2.1.2. Audio Recording

The analogue audio signal was sampled using a USB audio adapter (EW3751 R1, Ewent, Geleen, The Netherlands) with a measured frequency range of up to 44,100 Hz (human audible spectrum ~20–20 kHz) [23] and was connected to a laptop (XPS 13 9350, Dell, Round Rock, TX, USA). The audio signal was recorded with Audacity (version 2.3.2, The Audacity Team) in WAV-file format with a Sample Format of 32-bit float and a Sample Rate of 44,100 Hz and with microphone sensitivity set to 20%. A total of 2583 recordings were processed in this study.

#### 2.1.3. Data Analysis

**Figure 2:**
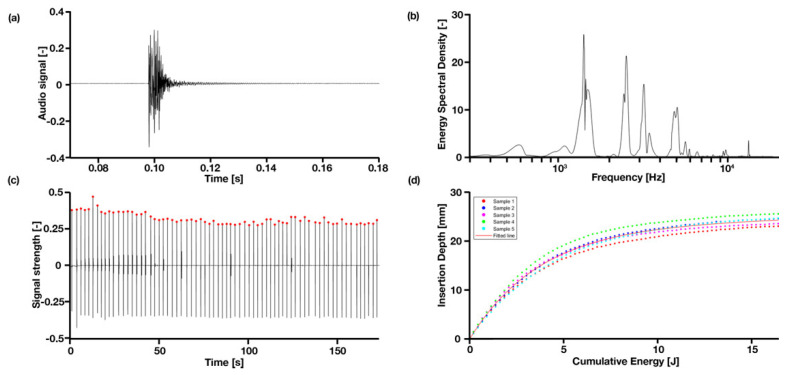
Typical hammer blow in time and frequency domain: (**a**) is the original audio signal; and (**b**) is the signal converted to ESD; (**c**) a complete set of hammer blows in signal intensity over time; (**d**) shows the cumulative energy over the insertion depth, caused by the hammer for the larger broach, well-fixated condition.

Data was analyzed using Matlab (version R2019b, MathWorks, Natick, MA, USA). The WAV audio format has a unit that is relative to the maximum measurable intensity, which is set to 1. The sample WAV file was split into separate hammer blows of 0.2 s with a bandwidth per peak of 300 Hz, from which the Fast Fourier Transform (FFT), a peak frequency search, was performed and subsequently the Energy Spectral Density (ESD) were calculated in Matlab as |fft(h)|^2^. Peaks were found using findpeaks in Matlab and within the function, MinPeakHeight and MinPeakDistance were determined by trial and error until we got the most reliable result for all experiment. The frequency peaks and their energy were analyzed, as well as the insertion depth and cumulative energy, defined as the cumulative hammer energy added to the system during the experiment. A typical hammer blow in time and frequency domain is presented in Figure 2a,b. Statistical significance in correlating peak frequency and insertion depth was performed in GraphPad Prism (Prism 8, San Diego, CA, USA) using a simple linear regression curve fit.

### 2.2. In Vitro Experiments

To test the BIMS and its working principle, a simplified bone model and implant model consisting of a simplified broach and connected handle were made. We refer ‘implant’ generally to the device used in hip arthroplasties; while ‘broach’ refers specifically to the ‘stem’ that gets inserted into the femur.

#### 2.2.1. Custom Implant Model

The implant model consists of a broach and handle shown in Figure 1a. The steel (Blankstaal S235JRG2C+C/SH 18 mm rond, Kloeckner Metals ODS Nederland, Barendrecht, The Netherlands) broach was simplified as a round wedge with a tip diameter of 7 mm, gradually increasing until 18 mm over 130 mm from the tip. Another thinner broach was also made, increasing from 7 mm to 15 mm over 130 mm to obtain an undersized condition with less fixation than the thicker broach at the same insertion depth. The dimensions were based approximately on two of the most-commonly-used sizes, 10 and 11, of the Corail broaches of Depuy Synthes of Johnson & Johnson. The steel (Platstaf 30 × 8 mm S 235 JR G2, Kloeckner Metals ODS Nederland, Barendrecht, The Netherlands) handle was simplified as two flat bars with a cross-section of 30 × 8 welded together perpendicularly, giving a total length of 255 mm. The broach and handle are connected with M4 threading.

#### 2.2.2. Custom Bone Model

The dimensions of the bone were comparable to a typical femur [24]. The bone model in Figure 1b was made from two materials: the cortical bone of the femur was modelled as rod of pine (Pinus sylvestris) wood (Grenen ronde stock 70% PEFC 28 × 2700, Pontmeyer, Zaandam, The Netherlands) with a length of 400 mm and an outer diameter of 28 mm. In this rod, a hole with a diameter of 15 mm and a depth of 200 mm was made and filled with polyurethane (PUR) foam (Precit montageschuim 30, Hornbach Baumarkt AG, Bornheim, Germany) to represent the trabecular bone. Wood was chosen as a repeatable in vitro material as Sawbone for instance is not feasible for large sample numbers. Furthermore, they exhibit similar mechanical properties for sawing/cutting during surgery. Properties of the materials used in the bone model were referred from CES EduPack (Granta Design Limited, Cambridge, United Kingdom) [25], which suggested a pine wood elastic modulus of 12,000–14,000 MPa and 0.33–665 MPa for PUR foam. The PUR foam was sprayed into the cavity until it was filled to the top and let set for a duration of at least 14 h. A hole of 100 mm deep and a diameter of 8.5 mm was reamed in the PUR foam to model the medullary cavity and to guide the broach into the foam. A set of 5 bones per experimental group was produced. The bone model was clamped in a silicone (SR1040 Siloconen gietrubber Platinum 40, Resion Resin Technology, Moordrecht, The Netherlands) frame, representing the soft tissue of the upper leg. This frame was secured in a vice to prevent movement.

#### 2.2.3. Experiment Setup

The setup is shown in Figure 1c,d, consisting of the microphone attached to the handle, the implant model inserted into the bone model, the bone model clamped with two vices and a swing hammer that hits the handle top. The vice was tightened until the bone model did not move visibly while hammering. The starting insertion depth was 65 mm, as the broach got to this depth without resistance. The hammer (Vuishamer 1500 g, Picard, Wuppertal, Germany) weighed 1.5 kg and could be released from several fixed positions to keep the exerted energy constant. A digital caliper was used to measure the insertion depth. The force required for removing the broach was measured with a Newton meter.

### 2.3. Experiments

To determine if there is a difference in frequency spectra between an intact fixated model (good fit), an intact loose model (non-optimum, slightly undersized broach), and a broken fixated model (crack/damage in bone), three experiments were conducted on the set of five identical custom bone models. Testing was done in three experiments: an insertion experiment, a fixation experiment, and a fracture experiment.

#### 2.3.1. Insertion Experiment

The larger-sized broach was pushed into the bone model for 65 mm first, before hammer blows were applied. The swing hammer was set to apply an energy of approximately 0.24 J per blow (neglecting friction in the hinge and other losses with simple estimate of the potential energy equation E_p_ = mgh) by swinging from a relative height of 16 mm until the broach advanced to the “end point”. This amount was predetermined not to cause fractures. In testing, the number of hammer blows needed for the insertion depth to change less than 0.1 mm per hammer blow in five consecutive blows was determined to be approximately 70. This number was used for the insertion experiment in all samples. At this point, we consider the broach fixated. In actual surgery, the surgeon would determine this “end point” pre-operative templating on X-ray images in combination with the chosen broach size. The surgeon has the freedom to fine tune the actual depth by a few mm if needed. After each hammer blow, the insertion depth was measured. After the last hammer blow, the extraction force to pull out the broach from the bone model in the axial direction was measured.

#### 2.3.2. Fixation Experiment

The smaller, undersized broach with a tapered end was used. Testing showed that after approximately 20 hammer blows, the smaller broach reached the same insertion depth as the previous, larger-sized broach. Therefore, 20 blows are applied for the fixation experiment and again, the extraction force was measured.

#### 2.3.3. Fracture Experiment

A fracture with a length of 50 mm and 1 mm width was sawn in each of the five bone models. This length was chosen after fracturing several bone models and an average fracture length was obtained. The large broach was used for this experiment. Testing showed that the final insertion depth was reached after approximately 100 hammer blows and this number was used in the fracture experiment. Another set of five bone models (without a pre-sawn crack) were used to compare fracture by hammering until fracture occurs. If no fracture occurred, then the energies were increased until an approximate 1.9 J maximum by dropping the hammer from a relative height of 132 mm.

### 2.4. Pilot Trial in Cadaver Model

To demonstrate the appropriateness of this setup in clinical settings, we also performed a trial on a cadaver hip arthroplasty during a training session. The study was carried out in Erasmus Medical Centre, Rotterdam, The Netherlands. The acoustic microphone was attached to the surgical handle of the Corail implant (Depuy Synthes, Johnson & Johnson, Raynham, MA, USA). The setup is shown in Supplementary Materials Figure S1.

### 2.5. Eigen Frequency Estimation

The simplified model including the handle and broach allowed an analytical estimation of the Eigen frequencies and frequency shifts to help interpret and compare the measured results. For these calculations, the implant model was simplified as a rectangular beam with consistent cross-section (30 × 8 mm) and clamped-free boundary conditions. The implant model length *L* from the broach handle was 350 mm at the starting point *s* and 325 mm at the end point *e*. The implant model material was modelled as steel with a Young’s modulus *E* of 210 MPa and a density *ρ* of 7850 kg/m^3^. The Eigen frequencies *ω* per mode *i* for a clamped-free beam, with an area moment of inertia *L* and mass per unit length *μ* is calculated by [26,27]:

(1) $$\omega_{i}={\left(\left(i-\frac{1}{2}\right)\pi \right)}^{2}\frac{1}{L^{2}}\;\sqrt{\frac{EI}{\mu }}$$

The Eigen frequencies were calculated for the first four modes in the direction of the width and height of the beam, defined as the x- and y-direction, respectively, and are shown in Table 1. The height of these peaks is defined as the peak energy and their frequencies as the peak frequency. The cumulative energy is defined as the sum of the hammering energy added to the system. Based on these “ballpark” estimations (granted, the boundary conditions are different), we used them as a guide to select the four peaks in experimental data for analysis.

### 2.6. Statistical Analyses

Firstly, a simple linear regression analysis was performed at each frequency peak to determine whether there is a statistically significant increase in the Eigen frequencies with insertion depth using Prism 8 statistical package (GraphPad, San Diego, CA, USA). When testing the null hypothesis of a zero slope, a *p*-value of < 0.05 suggests a non-zero linear slope, which reflects a (increasing) shift. Secondly, an exponential growth nonlinear regression analysis was performed on peak energy values to determine the ‘convergence’ of the data, as an indicator of implant fixation using $y=y_{0}\exp\left(kx\right)$, where *y*_0_ is the *y*-value when *x* (time) is zero; *k* is the rate constant (no slope as dotted lines in Figures in Section 3.3, Section 3.4 and Section 3.5 were plotted if the trend appeared weak). The R^2^ “goodness of fit” coefficient was used as a quantifier of the scatter (we propose a higher value implies a higher degree of fixation). Analysis was also performed with Prism 8. Outlier datapoints originated form this that represent noise or other factors (e.g., too low of a peak) that were removed or cropped by the axis limits. An F-test was used in the Prism to compare variances between the regression fits of Figures in Section 3.3, Section 3.4 and Section 3.5 (i.e., large vs. small, large vs. fractured and small vs. fractured cases) for each peak.

## 3. Results

In this paper, we investigated the acoustic emissions caused by hammering on the hip replacement broach handle. Three experiments were performed to investigate the effect of AEs from broach insertion characteristics, the fixation quality with a larger and smaller broach, and fractured samples.

### 3.1. General Observations

When hammered into the bone model, the simplified implant model produced frequency bands centered around four main resonant peaks, namely 1400 Hz, 2500 Hz, 3150 Hz and 4700 Hz, with a width of approximately 300 Hz. A typical hammer blow in raw audio signal is presented in Figure 2a in the time domain and (b) the frequency domain, and a whole set of hammer blows are shown in Figure 2c.

### 3.2. Energy Spectral Density

**Figure 3:**
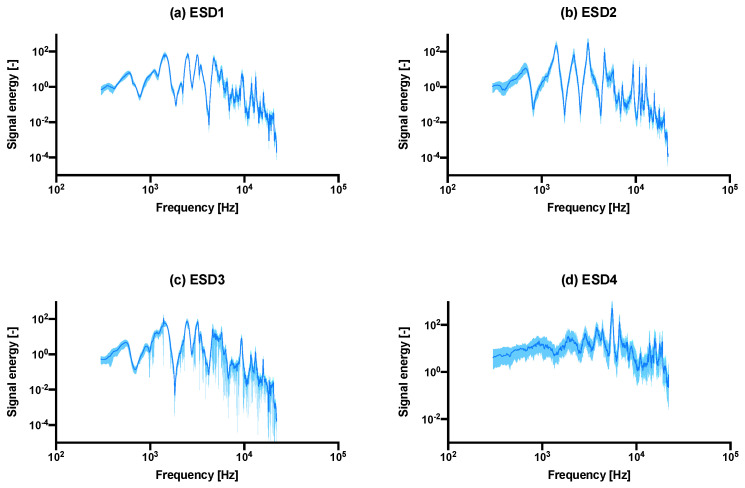
The median ± interquartile range (shaded) ESD plots comparison between the (**a**) last 20 hammer blows of all intact samples combined into one graph, vs. (**b**) the undersized/under-fitted broach, the (**c**) fractured samples, as well as the ESD recording from a pilot cadaver study using actual Depuy Synthes Corail components shown in (**d**). Interquartile range is shown instead of standard deviation as the distribution was asymmetrical. The same graph with a linear *y*-axis is shown in Supplementary Materials Figure S2.

The analyzed ESD plots shown in Figure 3 compare the in vitro bench top setup (a–c) versus an in situ cadaver study (d). The main observable peaks appeared to be consistent with each other, in terms of locations, although the peak energy heights of the non-fixated samples (b) are much greater than the well fixated group. In terms of determining fracture of the bone (c), it is difficult to determine (if at all), whether a fracture is present in the bone.

The number of significant peaks observed in the in vitro study was four, while the in situ study showed small peaks at approximately 1900 Hz, 2900 Hz, 3850 Hz, 4400 Hz, 6650 Hz and 15 kHz. An exceptionally high peak was observed at around 5550 Hz. The absolute value of signal energy was also much greater for the cadaver study and presumably, as was noted in the experiment section, this was due to the fact that the hammer blows exerted by the surgeon are greater than the controlled hammer blows from the rig. Despite these differences, the peaks recorded from both in vitro and in situ studies appeared in the same order of magnitude (in the range of 10^3^–10^4^ Hz), even though there are differences in materials and geometry.

### 3.3. Insertion Experiment

**Figure 4:**
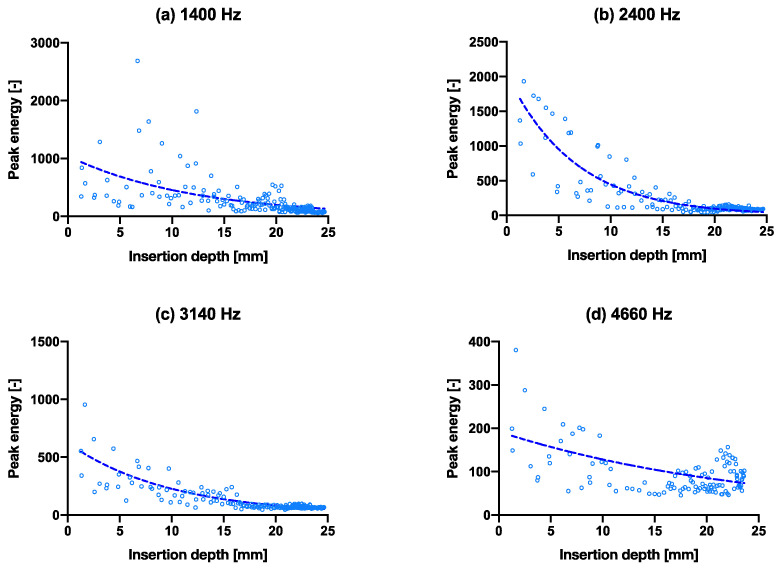
Capability of predicting insertion depth with frequencies by peak energy vs. insertion depth through the course of inserting the broach into the bone. (**a**–**d**) are the isolated peak energies (larger broach condition) for the four main peak frequencies at 1400 Hz, 2400 Hz, 3140 Hz and 4660 Hz, respectively, with exponential trend lines. The same graphs with hammer blows on the *x*-axis are shown in Supplementary Materials Figure S3.

Figure 2d shows a positive correlation between the insertion depth and cumulative energy—the same amount of energy is given in each hammer strike, however, as can be observed, the initial insertion depth increases linearly, until approximately 10 mm deep, then slowly flattens to a plateau in the end. All samples follow the same pattern. The convergence is observable in Figure 4 that all four peak energies from the ESD suggest it may be possible to use as an indicator to predict the insertion depth for a controlled energy applied with the hammer. The R^2^ values of the exponential fit for resonant peaks 1–4 are 0.31, 0.78, 0.70 and 0.26, respectively (Table 2). An upwards shift in peak frequency trend (significantly non-zero slope with *p* < 0.0004–0.0001) was observed for the 3rd and 4th peaks in both the smaller and larger broach, when inserted further into the bone model (Table 3).

### 3.4. Fixation Experiment

**Figure 5:**
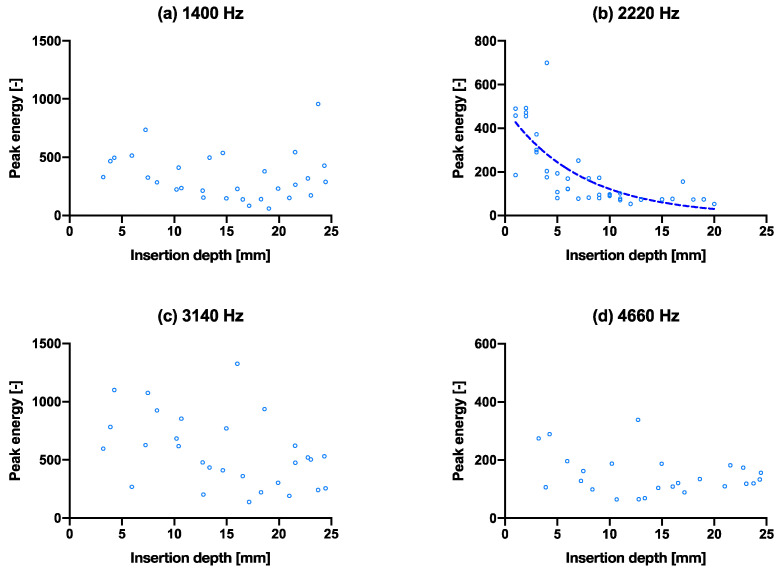
Capability of predicting fixation quality using the spread of data in peak energy vs. insertion depth. (**a**–**d**) are the isolated peak energies (smaller broach condition) for the four main peak frequencies at 1400 Hz, 2220 Hz, 3140 Hz and 4660 Hz, respectively, with exponential tren lines. The same graphs with hammer blows on the *x*-axis are shown in Supplementary Materials Figure S4. Fracture experiment.

The same experiment with the above insertion experiment was performed with a slightly smaller/thinner broach to simulate an undersized implant, as shown in Figure 5. The poorer fixation showed no- (a, c, d) or poor convergence (b) of the peak energies even as the stem went further into the bone model. The broach was also extracted after full insertion. The smaller broach extraction force was recorded at 60 ± 10 N, while the larger broach was recorded at 210 ± 40 N. The extraction force for the large broach due to the tighter fit is higher and is thus considered more (adequately) fixated than the smaller broach. The R^2^ values of the exponential fit for resonant peaks 1–4 are 0.12, 0.51, 0.20 and 0.17, respectively (Table 2), which are all lower than that of the larger broach.

### 3.5. Fixation Experiment

**Figure 6:**
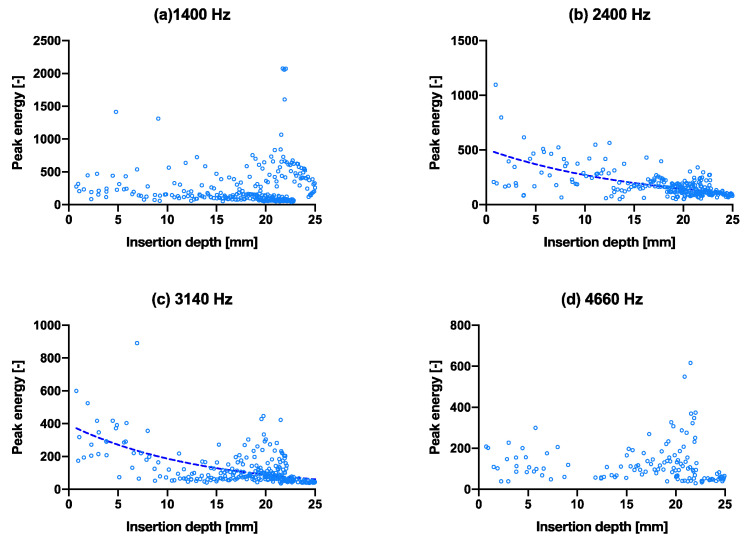
(**a**–**d**) are the isolated peak energies of each hammer blow, plotted as peak energy vs. insertion depth (fractured bone model condition) for the four main peak frequencies at 1400 Hz, 2400 Hz, 3140 Hz and 4660 Hz, respectively, with exponential trend lines. The same graphs with hammer blows on the *x*-axis are shown in Supplementary Materials Figure S5.

The deliberately created crack in the bone model appeared to show similar resonant peaks (height, width and position), in Figure 3a,c, and from the isolated peak energy charts in Figure 6a–d, they appear to show a varied degree of convergence, with the exception of the fourth peak (d). This is perhaps due to the small magnitude of the peak energy as observed in Figure 3c. The peak energies were shown at the same peak frequencies as per previous groups. Overall, it is difficult to distinguish between the non-fractured and fractured groups. For the fractured bone condition, the R^2^ values of the exponential fit for resonant peaks 1–4 are 0.00, 0.44, 0.36 and 0.02, respectively (Table 4). These values are all lower compared to the larger broach; however, the 3rd and 4th peak fittings are higher than that of the smaller broach (Table 2). Notably, peak 3 was found to be the only statistically similar regression curves between the large broach and fractured cases (results presented in Supplementary Materials Table S1). All other curves were found to be statistically significantly different enough between each other.

### 3.6. Cadaver Pilot Trial

To investigate the extent to which the results of the in vitro experiments were related to a real THA, the in situ results of the cadaver study were compared. We assumed an estimated impact energy of 1.75 J exerted by the surgeon, following the calculations proposed in the literature [22], with the mode of 3.5 kN as the average hammering force, which we suggest is a reasonable approximation, at least to the order of magnitude. While the exact insertion depth of the cadaver study was unknown, a rough approximation of 20 mm can be made. The broach inserted was a standard size nine, as specified in the Actis Surgical Technique [28]. In total, 59 blows were needed to hammer the broach into its end position, giving an estimated cumulative energy of approximately 103.25 J—which is about an order of magnitude lower when compared to the bone model (~12 J). Table 4 shows the differences between the in vitro and the in situ trial. The ESD of the cadaver study in Figure 3d shows different peaks (two main peaks as opposed to four) than the in vitro models (a–c), with different spread and peak magnitudes (1–6 times greater). There were two main peaks with several smaller peaks, instead of four main peaks.

## 4. Discussion

We performed experiments with two broach models, a ‘normal’ condition and an undersized condition, and two bone models, non-fractured and fractured. The objectives were to identify differences that could help determine the insertion depth of the broach into the bone through controlled hammering, the differences between a well-fixated and an under-fixated broach, and finally, the prediction of an imminent fracture due to over insertion and/or excessive hammering force. In general, the bone and broach models provided us with the ability to carry out the in vitro experiments consistently. The variance in data distribution may be caused by the variation in PU foam-filled core of the wooden bone model. In both the in vitro and in situ study, the contact microphone recorded a remarkably low amount of ambient noise (e.g., talking, instruments/equipment bumping into each other) in relation to the hammer blow, shown as gaps in Figure 2c, also compared to, for example, ambient audio recording [19], which suggests that it can be a useful tool in the OR and not just limited to recording THA acoustic emissions. In all studies, the frequency peaks are clearly visible and can be isolated to analyze, for example, the peak energy. The contact microphone used is mountable on the surgical handle and the vibration is transmitted without having to travel through air. In addition, the microphone is not invasive and is easy to place. Future designs can investigate a wireless solution.

The bone model was designed this way so that batches of them can be made consistently; artificial bone (vs. animal/cadaver bone) provides more consistency (although inter-specimen variability still exists with the PU foam); and we expect the principles of the vibrations to be comparable between the model and actual bones, although the frequencies might differ. Additionally, PUR foam shares similar structure and compressive strength with trabecular bone [21,25], so we expect a similar response. Granted, the absolute values of frequencies, for example, may not be the same, however, the general trends may still be similar. Alternative bone models such as Sawbones may provide better consistencies, however, this study focuses on proposing a diagnostic technique with an interest in process and model variation rather than characterizing the absolute values of a highly standardized synthetic model. An effort was made to source hard wood, however this was unavailable. That said, overall the material was comparable to Kutz’s report [21] to real bone. Regardless, we suggest that the results would still be comparable in the end, as overall the properties are still in the same orders of magnitude. Furthermore, we encased the wooden profiles in silicone to simulate soft tissues with the soft tissue boundary conditions in mind. When performing a frequency analysis during testing, the material properties that determine the Eigen frequency of the bone model could be considered.

To use the peak energies at Eigen-frequency bands as a tool to measure insertion depth, this was possible by observing the gradually increasing trend in resonant frequency shift upwards—which is expected when considering this as a fixed-free cantilever beam where the effective free end becomes shorter. The Eigen-frequencies calculated by hand overestimated the frequency response by an order of magnitude, most likely due to the boundary conditions and geometries used in the calculations. This is one of the limitations of this study, and in future, this can be improved by using, for example, finite element methods to obtain a better estimate. The upward frequency shift was visible in the third and the fourth peaks (Table 3 for significantly nonzero slopes where *p* < 0.05), in the order of ~100 Hz from the beginning to the end insertion over approximately 25 mm. This increase would most likely increase further with a real broach, as the change in insertion distance is much greater. It would be possible then to predict insertion depth, if the input force/energy is known or controllable, an extrapolation could be made from the first few hammer blows. For example, taking the peak energies (2nd peak) recorded in the first experiment. A logarithmic equation was obtained for Supplementary Materials Figure S6, with insertion depth (*ID*) in mm approximated as: $ID=-7.7\ln\frac{PE}{1532}$, where *PE* is the peak energy, working backwards from a given energy to estimate the insertion depth. It is important to note that this equation is based on our bone model, and will likely differ in real life, although we believe the logarithmic trend is still valid. Other factors that may influence the insertion depth include the surgeon’s hammering force and bone quality. This supports our original hypothesis that cumulative energy can be used as a parameter for insertion depth prediction. The ability to predict insertion depth would therefore increase the fitting accuracy and prevent over-insertion.

**Figure 7:**
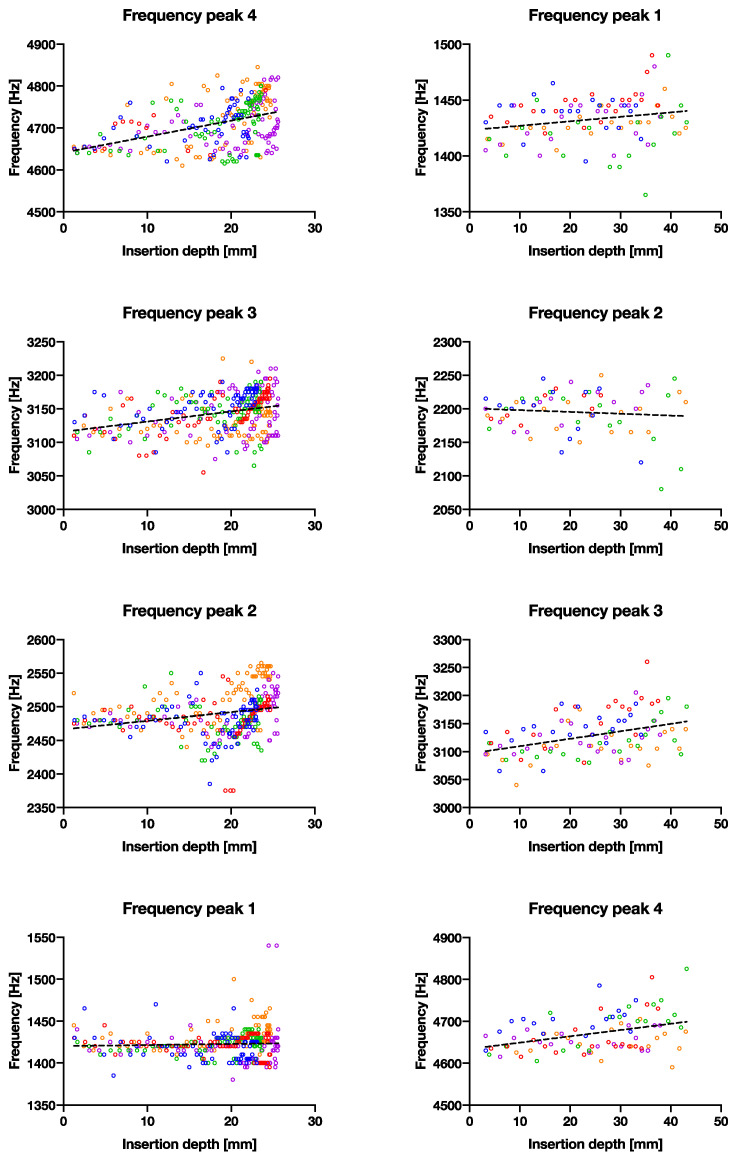
The correlation between recorded frequency vs. broach insertion depth with linear trend lines. Left column shows the larger broach with good fixation and right column shows the smaller broach with poor fixation. Each row represents the four main frequency peaks observed.

With fixation, we hypothesized that a difference is detectable between a well-fitted and an under-sized broach. The main difference identified here is that peaks 1 and 3 appeared to be greater (more than double) than that of a well-fixated broach (Figure 3a at ±100 vs. (b) at ±200–300, other peaks at ±100). Presumably, a looser fit dampens the vibrations less in an idealized free-fixed beam. The resonant frequencies (peaks 3 and 4) also shifted upwards significantly for both larger and smaller broaches (*p* < 0.0004–0.0001), as the broach is inserted further into the bone model (Figure 7). On the contrary, the consistency of the peak energies converged less between a poorly fixated and a well-fixated model (Figure 5 vs. Figure 4) as seen in the goodness of fit (R^2^) between the two datasets in Table 2. We propose that the higher the R2 may suggest lower scatter and implies a higher degree of convergence/consistency of the frequencies, suggesting more consistency of the implant being fixated into the femur. This finding was previously unreported in the literature and in future the R^2^ value could be used as an indicator/threshold to determine the fixation quality.

The peaks also appeared to differ in location and spacing, and thus, if the frequency patterns are known for a well-fixed implant, then it should be possible to identify under-sized or inadequately fitted implants in a patient with an overall ESD view of the acoustic emissions. During THA, the measured values may differ from the reported values herein with the model, as human bone is harder than the wood model, the cumulative energy may be higher before the end point is reached (also depending on how the bone is reamed). The exact value of the peak energy also depends on age or bone density [29] and the measuring equipment and settings, as the amplitude of the audio file (.wav) is on a relative scale. Therefore, we suggest using a relative threshold for a more robust model to be used on clinical data.

In regard to fracture, it was difficult to predict imminent fracture in our study. A set of data is shown in Supplementary Materials Figure S7. This is the third peak energy graph for one of the samples. It can be observed that after the initial decrease in peak energies, the energy increased slowly as the hammer blows continued to insert the stem further into the bone. This small and steady increase was not as visible as the other non-fractured groups. Whether this small increment of peak energy is an indication of stable crack growth, is so far only conjecture and needs further examination. Other cracked samples were fractured much earlier in the test, so this trend was not observed. Only immediately after fracture can an increase in peak energy and scatter be observed (most probably due to decreased fixation), however this indicator would not be suitable to prevent fracture in our setup. While the convergence of the resonant energies in Figure 6 suggests lower convergence than the original case, it also suggests that a fractured case does not have clear trends to be used as a prediction marker.

A test carried out (Supplementary Materials Figure S8) to see what it takes to crack the sample suggest that the insertion depth eventually stops advancing with each hammer blow, and that the energy of each strike has to be increased to advance the broach further, until the bone model cracks (an example of a cracked sample is shown in Figure S1a). This raises the question as to whether a hammer force exists which is incapable of causing fractures, while achieving the desired fixation. At the same time, we could possibly use this concept to obtain the desired insertion depth by controlling the hammering force. While determining an insertion force threshold that will not cause fractures depends on several factors such as bone quality, cavity geometry etc., this method still requires further refinement. Therefore, we suggest the comparison of the relative change in sound and/or broach advancement with the previous few hits in further studies. In Figure 4, for example, when the energy becomes quasi constant and this may help overcome the (in)accuracy in detecting the insertion depth near the “end point”. We propose that a more realistic cracked bone model may also show different crack growth or propagation modes and whether a saw cut created herein is an appropriate representation of a fracture. Perhaps a more representative method would be to create the crack using a chisel, although the fracture length would be less consistent. For now, though, it is difficult to predict fracture. We speculate, regardless, that fracture risk would be higher under a well-fixated condition than an under-fixated condition.

In relation to the cadaver pilot study, the in situ model compared well in terms of giving a similar range of peak resonant frequencies. The cadaver study also demonstrated that the setup is suitable for use in the OR environment with minimal intrusion to the surgeon, although the data was only analyzed after the session, and furthermore, it was not possible to measure the insertion depth during the trial due to it interfering with the surgeon. The cumulative energy exerted by the surgeon was approximately an order of magnitude higher than that of the bone model (~10^2^ J vs. ~10^1^ J), and about ten times more energy was required to move the same distance when compared with the bone model. Even though this is a very rough estimate with limited sample size (n = 1), the peak energy and frequency trends cannot be inferred (in which the patterns would be different to the in vitro model), and this should give an idea on how the bone models used in this research compare to real bones in OR conditions in terms of energies needed to insert a stem. One should bear in mind that the way the broach handle connects to the broach may alter the acoustic emission. In real life situations, the frequency peaks may also be dependent on a multitude of parameters relating to the patient (e.g., bone properties, geometry) and the environment (e.g., boundary conditions). We propose that one would approach this from observing the relative changes in peak frequencies as an example (rising/falling/flat), instead of the absolute values.

Additionally, the extra time spent setting up and configuring the device would prove costly due to expensive operating room (OR) time [30]. So, the design of such AE measurement should take usage time into consideration. Furthermore, devices that can reduce OR time add value to the surgery—in this case, measuring AEs could help inserting the implant more efficiently while also minimizing the risk of bone fractures that need repairing. The use of vibration analysis in assessing broach and stem fixation and fracture detection can thus prove to be a powerful tool during THA, as well as an invaluable learning tool. As the experience of the surgeons plays a substantial role in the successful outcome of the surgery, it is necessary that they are adequately trained for this procedure. A device that gives surgeons the feedback on the position and fixation of the broach and imminent fractures could help new orthopedic surgeons develop the experience they need to perform the surgery more quickly.

To address the limitations in our study, in future, this set of experiments should be carried out on real bones and conditions to gather more representative data, and to quantify the variability inherent in biological materials. Despite experiments suggesting that peak frequencies can provide valuable information about insertion depth, follow up studies are needed to link the data of this study to data obtained during real surgery. However, the fracture case is likely causing more variability in peak frequencies and likely altering the Eigen-frequencies (so not directly comparable to Figure 3) due to changing boundary conditions, and therefore further studies should include a thorough analysis of the peak frequencies and relation to the growing fracture size. A finite element model can also be established to correlate the experiment results, as well as to investigate the effect of a varied hammering force and/or fracture sizes on the peak energy curves, to further draw conclusions regarding the use of peak energy as an indicator of fixation. Other limitations that should be addressed include the spread of the data, which should be controllable with an in silico-based approach, and the limited number of in situ replicates and the variation in the in vitro material. The ideal “end points” in real life should also be quantified to build up a database for comparison. Hammering sound recorded in the OR should be analyzed in real time to provide an objective reading for the surgeon. A simple, no-setup display of the data should also be studied. For use in actual surgeries though, the microphone setup also needs to be made wireless and serializable. Alternative sound recording methods could also be explored, such as with a regular microphone, however, background noise issues will need to be isolated. Regardless of the method of recording, measuring acoustic emissions from arthroplasty surgeries, including the shoulder and the knee, could eventually become a mainstream tool to analyze the quality of implantation, as well as potentially insertion, fixation and fracture prediction and prevention, forming the basis for assisted autonomous arthroplasty surgery. Eventually as a commercial product, we envisage an instrumented broach would measure the frequencies and forces of each hammer blow, then transmit to an external device that would estimate the optimal placement and fracture risk against a database with patient body parameters taken into consideration.

## 5. Conclusions

This paper explores the possibilities of using vibration analysis in arthroplasty surgeries in a user-friendly setup. While the earlier literature suggests that vibration analysis can offer considerable benefits such as insertion depth and fracture prediction, it is not yet implemented routinely as part of a standard procedure in a small or convenient setup for the OR. Insertion depth of the broach followed a predictable pattern with a known amount of energy applied at each hammer blow. Results suggest that the third and fourth resonant frequency during a hammer blow correlated with insertion depth. The fixation experiment showed that it is possible to identify the degree of fixation based on how well the recorded energy peaks converge. The fracture experiment showed that it is difficult to predict imminent fracture with the current conditions, as the resonance peaks appeared quite similar to the group without a simulated fracture in the model. Only after cracking did the peak energy increase back to the original magnitude at the start of hammering. With further refinements to the system, such as sterilization environment compatibility, and a display of the recorded data, can this eventually be implemented in clinical studies.

## Figures and Tables

**Table 1:** Analytical estimations of the Eigen frequencies of the broach at the starting and ending positions in x and y directions.

Mode *i*	ω_s,x,i_ (Hz)	ω_s,y,i_ (Hz)	ω_e,x,i_ (Hz)	ω_e,y,i_ (Hz)
1	241	902	279	105
2	2170	8120	2510	9420
3	6010	22,600	6980	26,200
4	11,800	44,200	13,700	51,300

**Table 2:** Curve-fitting of Figure 4, Figure 5 and Figure 6. The degree of convergence of the pitch consistency analogous to the goodness of fit (R^2^) may suggest fixation quality.

Nonlinear Regression	Peak Energy			
Figure	4a	4b	4c	4d
Exp. eqn. best-fit values	$y=1044e^{-0.0826x}$	$y=2041e^{-0.1519x}$	$y=624.1e^{-0.1009x}$	$y=192.6e^{-0.04061x}$
R^2^	0.3103	0.7783	0.6959	0.2554
Figure	5a	5b	5c	5d
Exp. eqn. best-fit values	$y=486.7e^{-0.01864x}$	$y=491.6e^{-0.1399x}$	$y=813.7e^{-0.02262x}$	$y=83.88e^{0.02983x}$
R^2^	0.1199	0.5147	0.1979	0.1682
Figure	6a	6b	6c	6d
Exp. eqn. best-fit values	$y=285.7e^{0.001743x}$	$y=505.9e^{-0.06232x}$	$y=393.7e^{-0.07446x}$	$y=145.3e^{-0.0145x}$
R^2^	0.0001	0.4357	0.3550	0.0201

**Table 3:** Simple linear regression of figure in the Section 4 frequency peaks 3 and 4 for both small and large broaches show correlating increasing pitch frequency with insertion depth (NS: *p* > 0.05, *: *p* ≤ 0.05, **: *p* ≤ 0.01, ***: *p* ≤ 0.001, ****: *p* ≤ 0.0001).

	(a) F. Peak 1	(a) F. Peak 2	(a) F. Peak 3	(a) F. Peak 4	(b) F. Peak 1	(b) F. Peak 2	(b) F. Peak 3	(b) F. Peak 4
R^2^	0.002108	0.05928	0.09989	0.1856	0.04461	0.008432	0.1517	0.1351
*p* value	0.3925	<0.0001	<0.0001	<0.0001	0.0359	0.453	<0.0001	0.0004
Deviation from zero?	NS	****	****	****	*	NS	****	***
Equation	Y = 0.1314X + 1420	Y = 1.273X + 2466	Y = 1.519X + 3116	Y = 3.812X + 4641	Y = 0.3982X + 1423	Y = −0.2705X + 2201	Y = 1.331X + 3096	Y = 1.500X + 4634

**Table 4:** Comparison between the in vitro bone model vs. in situ cadaver trial.

	Bone Model Insertion Experiment	Cadaver Trial
Hammer blows	50	59
Insertion depth [mm]	23	20
Energy per blow [J]	0.24	1.75
Cumulative energy [J]	12	103.25
Energy needed to advance broach [J/mm]	0.52	5.16

## Data Availability

The datasets generated and/or analyzed from this study are available from the corresponding author on reasonable request.

## Supplementary Materials

The following supporting information can be downloaded at: https://www.mdpi.com/article/10.3390/s22041609/s1, Figure S1: (a) Fracture caused by over-insertion of the broach. (b) The contact microphone attached to the Depuy Synthes Corail broach handle for the cadaver pilot study. (c) The experiment measuring the hammering impact during the cadaver pilot trial.; Figure S2: The same graph of Figure 3 presented with linear *y*-axis.; Figure S3: The same graph as Figure 4, with hammer blow on the *x*-axis. (a–d) are isolated peak energies of the larger broach condition for the four main peaks at 1400 Hz, 2400 Hz, 3140 Hz and 4660 Hz respectively. Figure S4: The same graph as Figure 5, with hammer blow on the *x*-axis. (a–d) are isolated peak energies of the larger broach condition for the four main peaks at 1400 Hz, 2400 Hz, 3140 Hz and 4660 Hz respectively. Figure S5: The same graph as Figure 6, with hammer blow on the *x*-axis. (a–d) are isolated peak energies of the larger broach condition for the four main peaks at 1400 Hz, 2400 Hz, 3140 Hz and 4660 Hz respectively. Figure S6: Convergence of peak energies (2nd peak) as the broach is inserted further into the bone. This graph is similar to Figure 4b, at 2400 Hz, or Supplementary Figure S3 (with peak energy vs. hammer blow instead). Figure S7: Peak energy shown here decrease initially, then slowly increases as the broach is hammered in further, until catastrophic failure of the bone, at which point, the peak energy shoots up to the initial position because in effect, the broach is not constrained by the bone. Figure S8: Insertion depth eventually plateaus with each hammer blow, and the energy has to be increased (red line) to advance the broach further, until a point where the bone fractures and the broach can be pushed in at much greater pace. Table S1: F-tests of all regressions from Figure 4, Figure 5 and Figure 6 comparing whether the fittings are statistically different. *p* value summary symbol meanings: ns (*p* > 0.05), (*p* ≤ 0.05), * (*p* ≤ 0.01), ** (*p* ≤ 0.001), *** (*p* ≤ 0.0001).

## References

[1] Landelijke Registratie Orthopedische Implantaten Number of Registered Hip Arthroplasties Per Year of Surgery (2007–2018) in the Lroi in April 2019. http://www.lroi-rapportage.nl/2-magazines-op-home-2019-numbers-registered-procedures-2007-2018.

[2] Monitoring Femoral Component Insertion in Cementless Total Hip Arthroplasty. http://citeseerx.ist.psu.edu/viewdoc/download?doi=10.1.1.506.6517&rep=rep1&type=pdf.

[3] Whitwell G., Brockett C.L., Young S., Stone M., Stewart T.D. (2013). Spectral analysis of the sound produced during femoral broaching and implant insertion in uncemented total hip arthroplasty. Proc. Inst. Mech. Eng. Part H J. Eng. Med..

[4] Pastrav L.C., Jaecques S.V.N., Jonkers I., Perre G.V.D., Mulier M. (2009). In vivo evaluation of a vibration analysis technique for the per-operative monitoring of the fixation of hip prostheses. J. Orthop. Surg. Res..

[5] Rashid M.S., Pullin R. (2014). The sound of orthopaedic surgery—the application of acoustic emission technology in orthopaedic surgery: A review. Eur. J. Orthop. Surg. Traumatol..

[6] Patrick A.J.F., Rodgers G.W., Hooper G.J., Woodfield T.B. (2017). Development and validation of an acoustic emission device to measure wear in total hip replacements in-vitro and in-vivo. Biomed. Signal Processing Control..

[7] Qi G., Mouchon W.P., Tan T.E. (2003). How much can a vibrational diagnostic tool reveal in total hip arthroplasty loosening?. Clin. Biomech..

[8] Oberst S., Baetz J., Campbell G., Lampe F., Lai J.C., Hoffmann N., Morlock M. (2018). Vibro-acoustic and non-linear analysis of cadaveric femoral bone impaction in cavity preparations. Int. J. Mech. Sci..

[9] Rodgers G.W., Welsh R., King L.J., Patrick A.J.F., Woodfield T.B., Hooper G.J. (2017). Signal processing and event detection of hip implant acoustic emissions. Control Eng. Pract..

[10] Pechon P.H.M., Pullin R., Eaton M.J., Jones S.A., Evans S. (2018). Acoustic emission technology can warn of impending iatrogenic femur fracture during femoral canal preparation for uncemented hip replacement. a cadaveric animal bone study. J. Med. Eng. Technol..

[11] Gueiral N., Nogueira E. (2011). Recent Advances in Arthroplasty.

[12] Mavrogordato M., Taylor M., Taylor A., Browne M. (2011). Realtime monitoring of progressive damage during loading of a simplified total hip stem construct using embedded acoustic emission sensors. Med. Eng. Phys..

[13] Mulier M., Pastrav C., van der Perre G. (2008). Per-operative vibration analysis: A valuable tool for defining correct stem insertion: Preliminary report. Ortop. Traumatol. Rehabil..

[14] Shibanuma N., Hata Y., Nishiyama T., Fujishiro T., Tateishi H., Kurosaka M. Determination of total hip arthroplasty stem stability by intraoperative measurement using an acoustic testing technique. Proceedings of the 54th Annual Meeting of the Orthopaedic Research Society.

[15] Poudrel A., Lomami H., Rosi G., Dubory A., Flouzat-Lachaniette C., Haiat G. Estimation of Cementless Femoral Stem Stability Using an Impact Hammer. https://hal.inria.fr/hal-03235369.

[16] Leuridan S., Goossens Q., Pastrav L., Mulier M., Desmet W., Vander Sloten J., Denis K. (2021). Development of an Instrument to Assess the Stability of Cementless Femoral Implants Using Vibration Analysis During Total Hip Arthroplasty. IEEE J. Transl. Eng. Health Med..

[17] Michel A., Bosc R., Meningaud J., Hernigou P., Haiat G. (2016). Assessing the Acetabular Cup Implant Primary Stability by Impact Analyses: A Cadaveric Study. PLoS ONE.

[18] Goossens Q., Leuridan S., Henyš P., Roosen J., Pastrav L., Mulier M., Desmet W., Denis K., Vander Sloten J. (2017). Development of an acoustic measurement protocol to monitor acetabular implant fixation in cementless total hip Arthroplasty: A preliminary study. Med. Eng. Phys..

[19] Goossens Q., Pastrav L., Roosen J., Mulier M., Desmet W., Vander Sloten J., Denis K. (2020). Acoustic analysis to monitor implant seating and early detect fractures in cementless THA: An in vivo study. J. Orthop. Res..

[20] Morohashi I., Iwase H., Kanda A., Sato T., Homma Y., Mogami A., Obayashi O., Kaneko K. (2017). Acoustic pattern evaluation during cementless hip arthroplasty surgery may be a new method for predicting complications. SICOTJ.

[21] Kutz M. (2003). Standard Handbook of Biomedical Engineering and Design.

[22] Crisman A., Yoder N., McCuskey M., Meneghini R., Cornwell P. (2007). Femoral Component Insertion Monitoring Using Human Cadaveric Specimens. Conference Proceedings of the Society for Experimental Mechanics Series, 01. https://citeseerx.ist.psu.edu/viewdoc/download?doi=10.1.1.529.3840&rep=rep1&type=pdf.

[23] Purves D., Augustine G.J., Fitzpatrick D., Katz L., LaMantia A.-S., McNamara J., Mark Williams S. (2001). The Audible Spectrum. Neuroscience.

[24] O’Connor J., Borges L., Duda F., da Cruz A. Growth and reabsorption in biological tissues. Proceedings of the XXXVI Ibero-Latin American Congress on Computational Methods in Engineering (cilamce2015).

[25] CES EduPack Software. https://www.ansys.com/products/materials/granta-edupack.

[26] Eigenfrequency Analysis. https://www.comsol.com/multiphysics/eigenfrequency-analysis.

[27] Blevins R.D., Blevins R.D. (2015). Natural Frequency of Beams. Formulas for Dynamics, Acoustics and Vibration.

[28] Actis Surgical Technique. http://synthes.vo.llnwd.net/o16/LLNWMB8/US%20Mobile/Synthes%20North%20America/Product%20Support%20Materials/Technique%20Guides/Actis%20Surgical%20Technique%20March%202018.pdf.

[29] Mosekilde L. (1989). Sex Differences in Age-Related Loss of Vertebral Trabecular Bone Mass and Structure—Biomechanical Consequences. Bone.

[30] Childers C.P., Maggard-Gibbons M. (2018). Understanding costs of care in the operating room. JAMA Surg..

