# Supplementary material for: Using Acoustic Vibrations as a Method for Implant Insertion Assessment in Total Hip Arthroplasty
Source: Sensors (Basel). 2022 Feb 18;22(4):1609. doi: 10.3390/s22041609 (PMC8877904; doi:10.3390/s22041609)
Supplement: Supplementary file 1 [file sensors-22-01609-s001.zip › sensors-1509225-Supplementary.pdf]

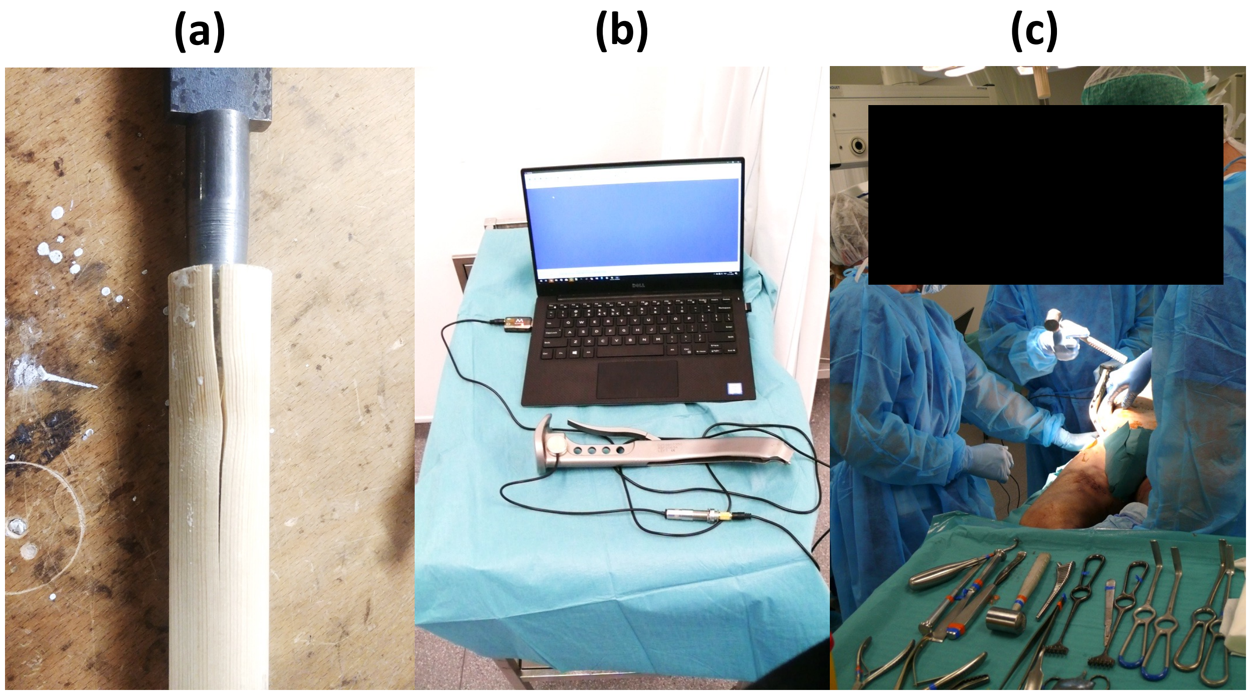

Figure S1. (a) Fracture caused by over-insertion of the broach. **(b)** The contact microphone attached to the Depuy Synthes Corail broach handle for the cadaver pilot study. **(c)** The experiment measuring the hammering impact during the cadaver pilot trial.

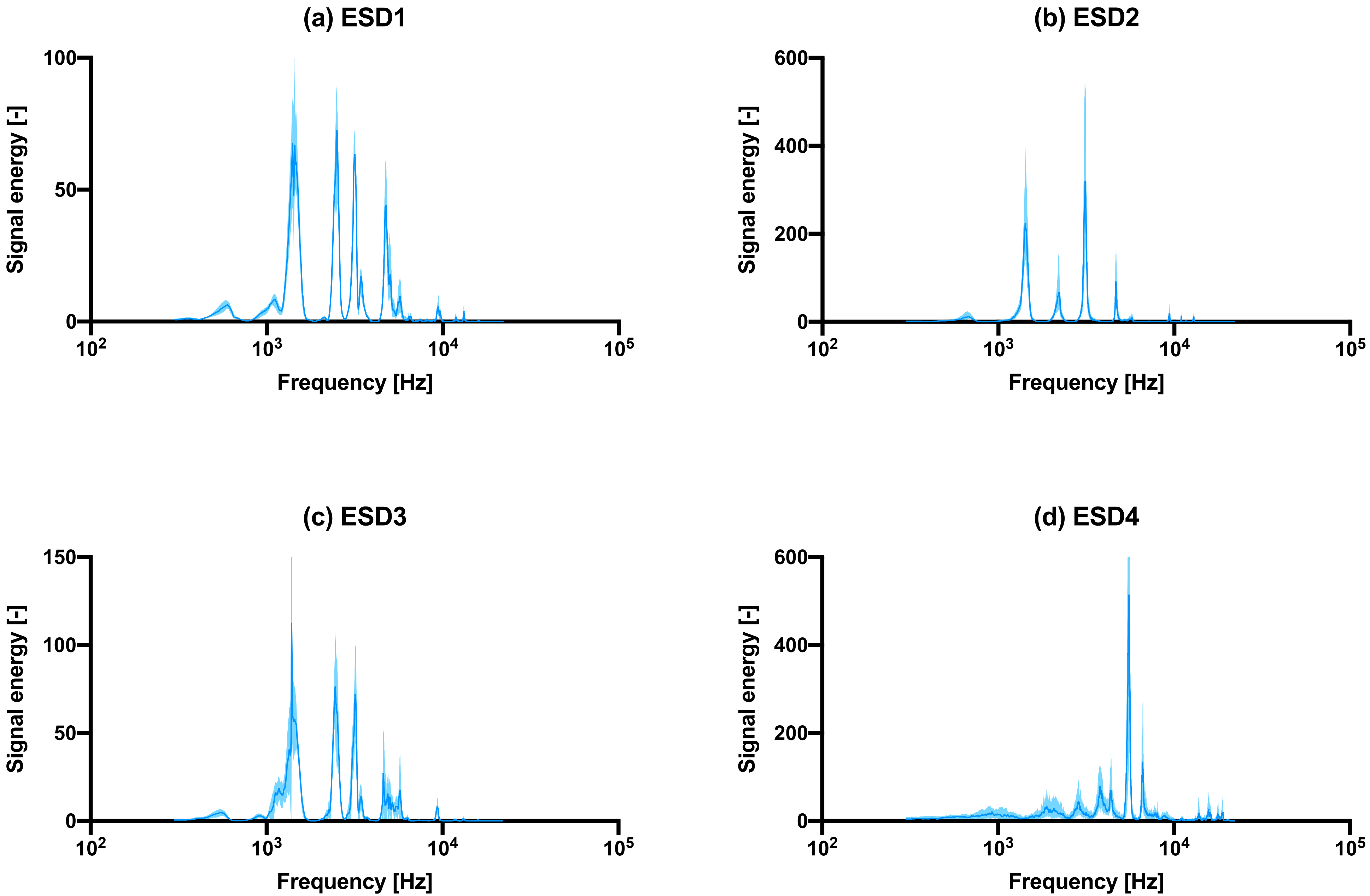

Figure S2. The same graph of Figure 3 presented with linear y-axis.

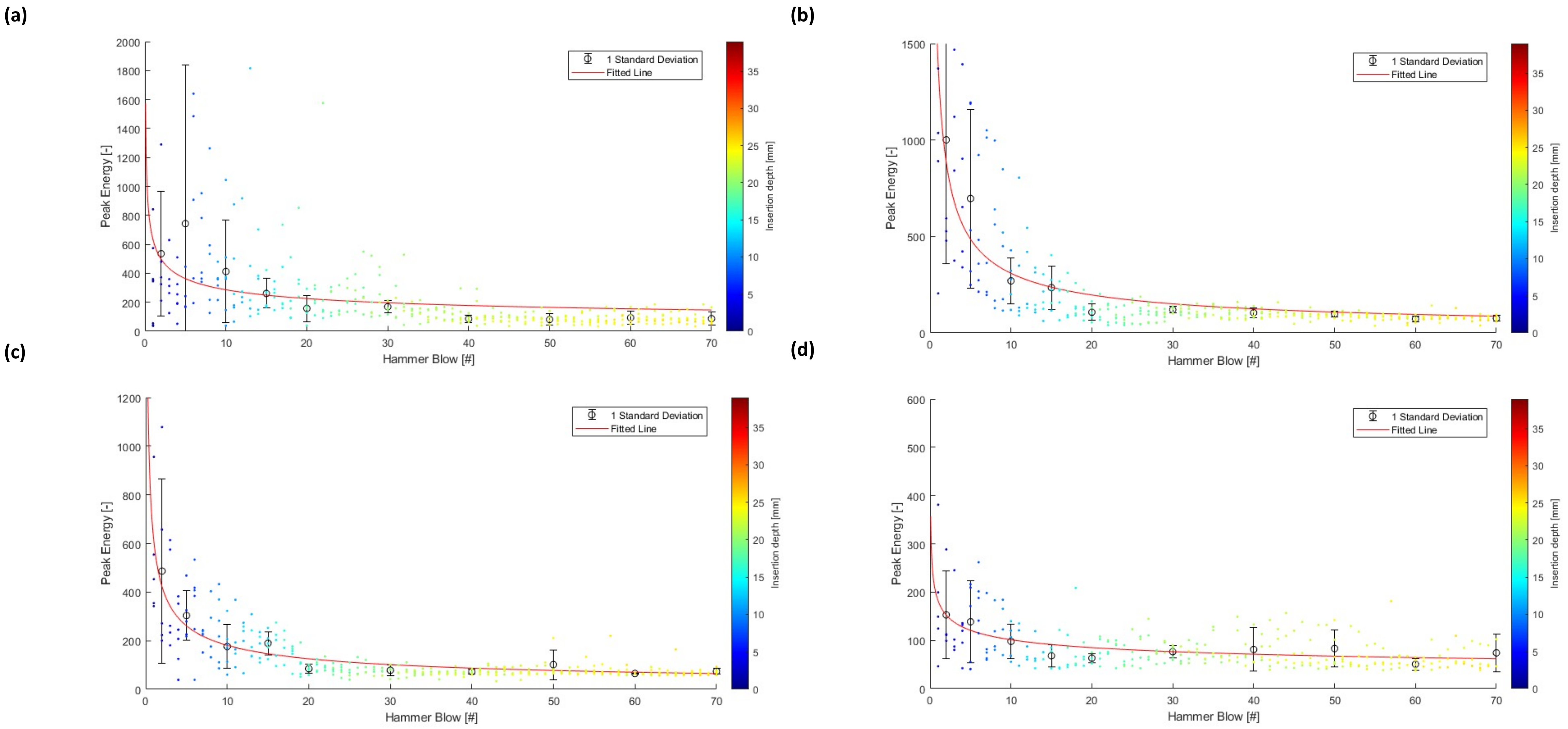

Figure S3. The same graph as Figure 4, with hammer blow on the x-axis. (a-d) are isolated peak energies of the larger broach condition for the four main peaks at 1400 Hz, 2400 Hz, 3140 Hz and 4660 Hz respectively.

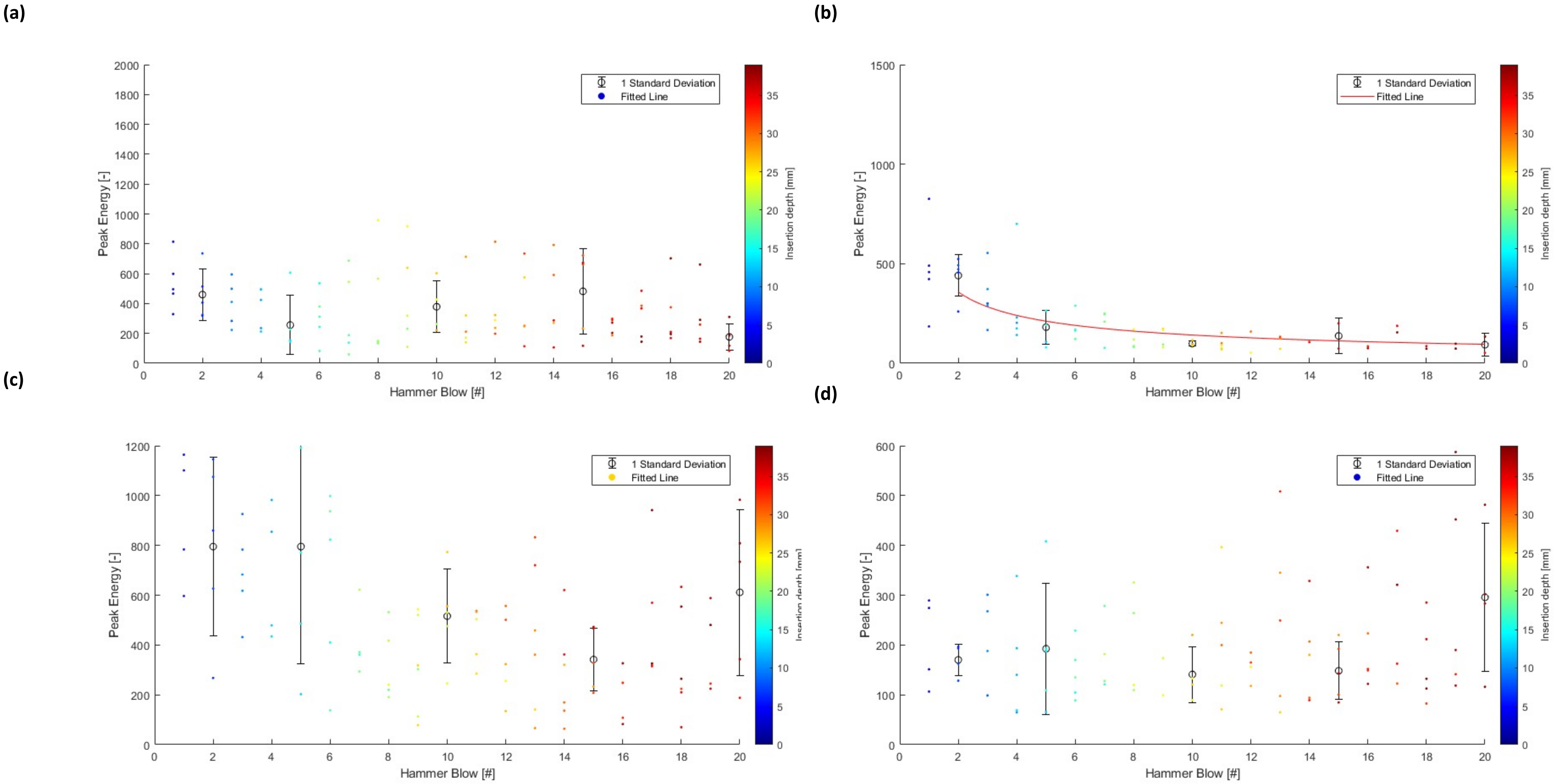

Figure S4. The same graph as Figure 5, with hammer blow on the x-axis. (a-d) are isolated peak energies of the larger broach condition for the four main peaks at 1400 Hz, 2400 Hz, 3140 Hz and 4660 Hz respectively.

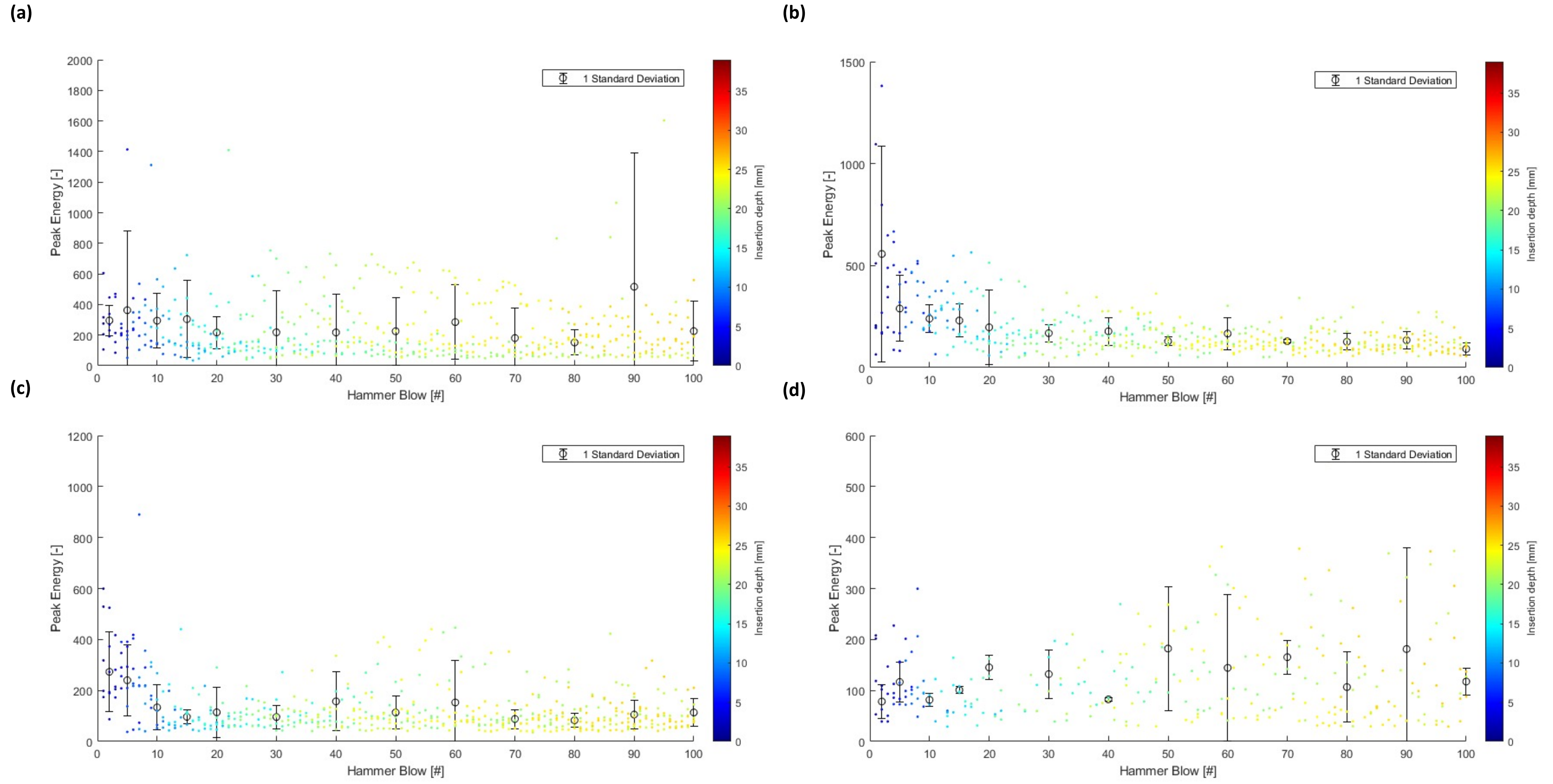

Figure S5. The same graph as **Figure 6**, with hammer blow on the x-axis. (a-d) are isolated peak energies of the larger broach condition for the four main peaks at 1400 Hz, 2400 Hz, 3140 Hz and 4660 Hz respectively.

Frequency Peak Energy around 2500 Hz

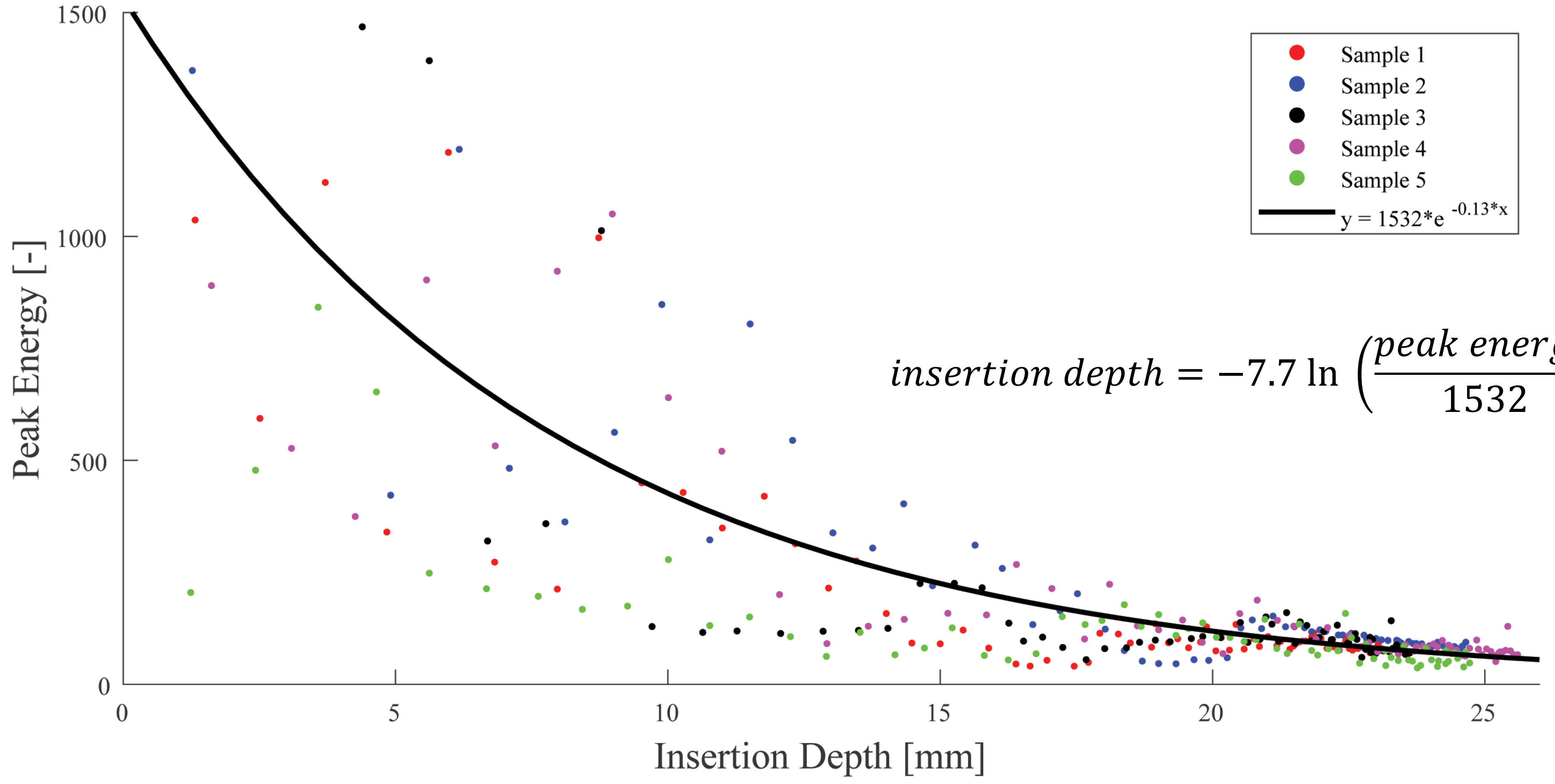

$$insertion\ depth = -7.7 \ln\left(\frac{peak\ energy}{1532}\right)$$

Figure S6. Convergence of peak energies (2nd peak) as the broach is inserted further into the bone. This graph is similar to Figure 4 (b), at 2400 Hz, or Supplementary Figure S3 (with peak energy vs. hammer blow instead).

Frequency Peak Energy around 3150 Hz

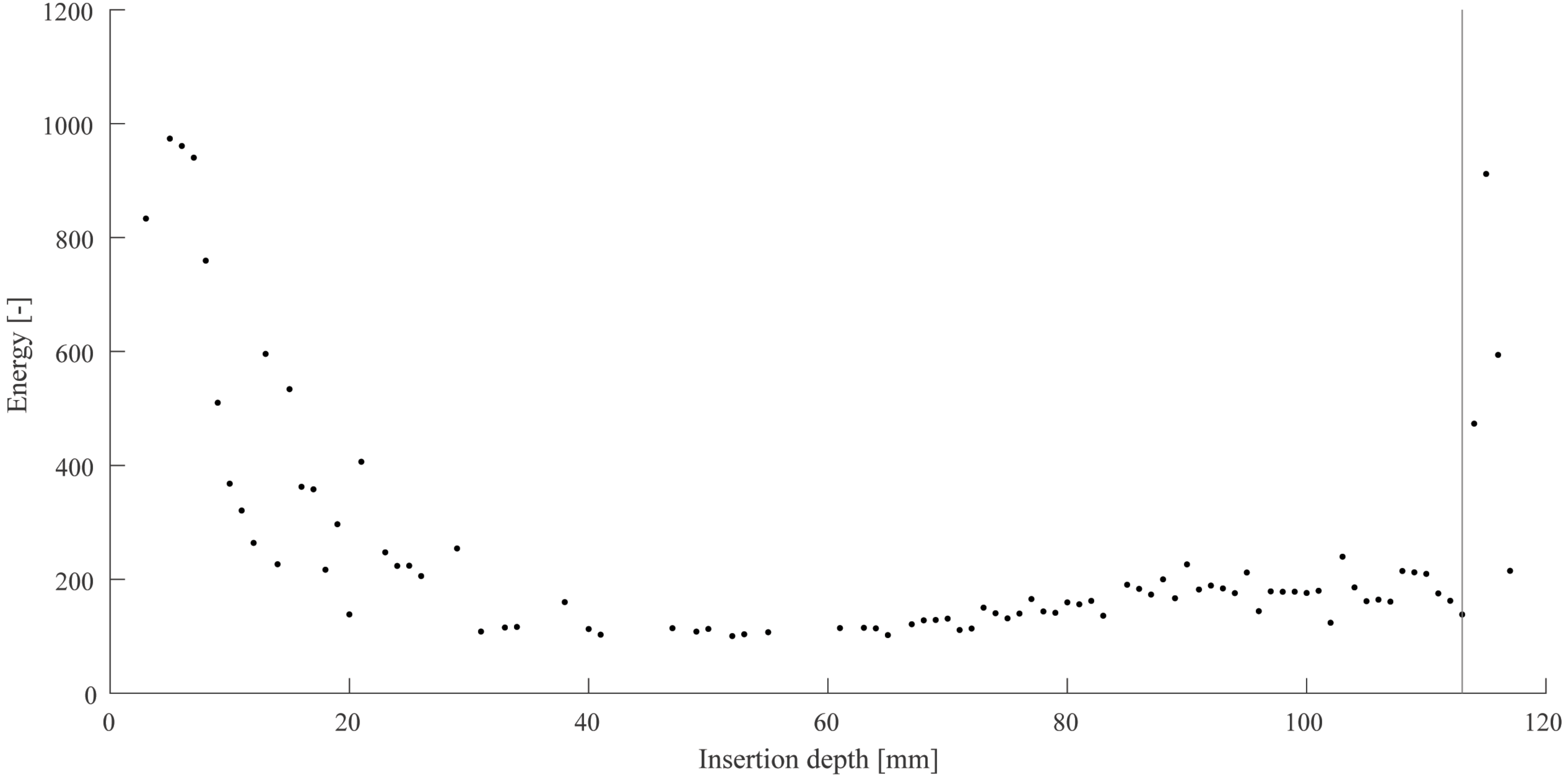

Figure S7. Peak energy shown here decrease initially, then slowly increases as the broach is hammered in further, until catastrophic failure of the bone, at which point, the peak energy shoots up to the initial position because in effect, the broach is not constrained by the bone.

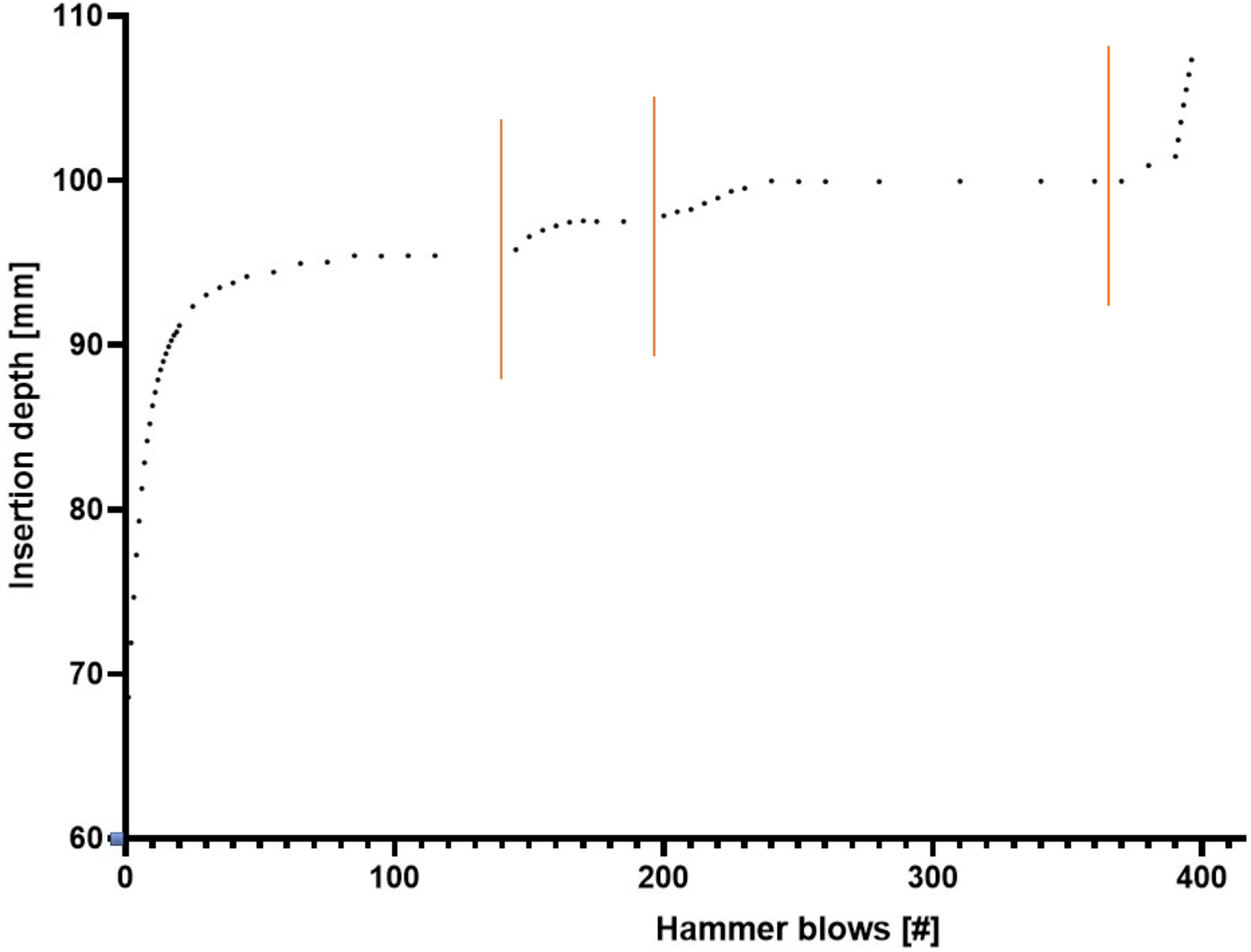

Figure S8. Insertion depth eventually plateaus with each hammer blow, and the energy has to be increased (red line) to advance the broach further, until a point where the bone fractures and the broach can be pushed in at much greater pace.

F test to compare variances	Peak 1		
	Large vs. small	Large vs. fractured	Small vs. fractured
F, DFn, Dfd	2.832, 279, 79	1.336, 279, 394	2.120, 394, 79
P value	<0.0001	0.0083	<0.0001
P value summary	****	**	****
Significantly different (P < 0.05)?	Yes	Yes	Yes
	Peak 2		
	Large vs. small	Large vs. fractured	Small vs. fractured
F, DFn, Dfd	4.381, 274, 54	7.252, 274, 370	1.655, 54, 370
P value	<0.0001	<0.0001	0.0081
P value summary	****	****	**
Significantly different (P < 0.05)?	Yes	Yes	Yes
	Peak 3		
	Large vs. small	Large vs. fractured	Small vs. fractured
F, DFn, Dfd	4.839, 78, 270	1.206, 270, 359	5.837, 78, 359
P value	<0.0001	0.0978	<0.0001
P value summary	****	ns	****
Significantly different (P < 0.05)?	Yes	No	Yes
	Peak 4		
	Large vs. small	Large vs. fractured	Small vs. fractured
F, DFn, Dfd	5.837, 68, 178	3.050, 168, 178	1.914, 68, 168
P value	<0.0001	<0.0001	0.0008
P value summary	****	****	***
Significantly different (P < 0.05)?	Yes	Yes	Yes

Supplementary Table S1. F-tests of all regressions from Figures 4-6 comparing whether the fittings are statistically different. P value summary symbol meanings: ns (P>0.05), * (P≤0.05), ** (P≤0.01), *** (P≤0.001), **** (P≤0.0001).
